# Long-Term Consumption of Purified Water Altered Amino Acid, Fatty Acid and Energy Metabolism in Livers of Rats

**DOI:** 10.3390/metabo14050289

**Published:** 2024-05-19

**Authors:** Jia Wang, Zhiqun Qiu, Hui Zeng, Yao Tan, Yujing Huang, Jiaohua Luo, Weiqun Shu

**Affiliations:** 1 Department of Medical English, College of Basic Medicine, Army Medical University, Chongqing 400038, China; awcm@tmmu.edu.cn; 2 Department of Environmental Hygiene, College of Preventive Medicine, Army Medical University, Chongqin 400038, China; qiuzhiqun@tmmu.edu.cn (Z.Q.); zenghui@tmmu.edu.cn (H.Z.); xiaoyue7122@tmmu.edu.cn (Y.T.); huangyujing@tmmu.edu.cn (Y.H.)

**Keywords:** purified water, low-mineral water, metabolism, metabolomics, negative nitrogen balance

## Abstract

The consumption of low-mineral water has been increasing worldwide. Drinking low-mineral water is associated with cardiovascular disease, osteopenia, and certain neurodegenerative diseases. However, the specific mechanism remains unclear. The liver metabolic alterations in rats induced by drinking purified water for 3 months were investigated with a metabolomics-based strategy. Compared with the tap water group, 74 metabolites were significantly changed in the purified water group (6 increased and 68 decreased), including 29 amino acids, 11 carbohydrates, 10 fatty acids, 7 short chain fatty acids (SCFAs), and 17 other biomolecules. Eight metabolic pathways were significantly changed, namely aminoacyl–tRNA biosynthesis; nitrogen metabolism; alanine, aspartate and glutamate metabolism; arginine and proline metabolism; histidine metabolism; biosynthesis of unsaturated fatty acids; butanoate metabolism; and glycine, serine and threonine metabolism. These changes suggested that consumption of purified water induced negative nitrogen balance, reduced expression of some polyunsaturated fatty acids and SCFAs, and disturbed energy metabolism in rats. These metabolic disturbances may contribute to low-mineral-water-associated health risks. The health risk of consuming low-mineral water requires attention.

## 1. Introduction

Due to worldwide population growth, industry development, water pollution, and climate change, water scarcity is a pervasive threat to society [1]. Since seawater constitutes more than 97% of total water resources, seawater desalination has turned out to be the most promising and efficient way to provide clean water [2]. The desalination of seawater and brackish groundwater via reverse osmosis (RO) provides water to some of the world’s most water-stressed cities [3]. The desalination capacity exceeded 100 million m3/d worldwide in 2020 [2]. What is more, RO also has become popular in household-scale water treatment and in the production of bottled water [3]. More than half (59%) of the commercial bottled water in the world is purified water [4]. RO systems remove not only impurities from water but also 92–99% of beneficial minerals including calcium, fluoride, and magnesium [5]. Besides desalinated seawater and bottled water, there is increasing use of recovered/recycled water, harvested rainwater, and point-of-use water [4]. Desalinated water, bottled purified water, recovered/recycled water, and point-of-use water have extremely low mineral contents. In addition, the natural water in many areas is soft or low in many minerals [6]. Therefore, more and more people have access to low-mineral water.

Desalinated or demineralized water that has not been further enriched with some minerals might not be appropriate for consumption [6]. Epidemiologic studies have documented an inverse relationship between the mineral content (or hardness) of water and cardiovascular disease [7,8]. The intake of low-mineral water may be associated with a higher risk of hip fracture in men [9], lower bone mineral content [10], certain neurodegenerative diseases [11], pre-term birth and low weight at birth [12], stunted height growth, increased dental caries in children [13], and some types of cancer [14,15]. Human intervention studies showed that drinking low-mineral water exacerbates lipid profile and raises homocystine (an independent cardiovascular disease biomarker) levels [16]. On the contrary, drinking natural mineral water may promote bone health, maintain cardiovascular function, improve lipid profile, aid weight management, and enhance overall well-being [17]. Animal studies also proved that the consumption of low mineral water leads to a higher risk of cardiovascular disease [16] and impaired bone quality [18]. Meanwhile, the consumption of natural mineral water may contribute to controlling blood lipid and glucose levels [19], improving glycemic control [20], and maintaining bone health in young rats with metabolic acidosis [21].

The long-term consumption of low-mineral water can cause harm to human health. However, the specific mechanism remains unclear. The study of alterations in cellular metabolic pathways is important to further understand the biological responses and disorders associated with drinking low-mineral water. Mass spectrometry-based metabolomics approaches can enable the detection and quantification of many thousands of metabolite features simultaneously [22]. To the best of our knowledge, no past study utilized a metabolomics-based strategy to explore the health effects of low-mineral water. Previous studies have proved that drinking low-mineral water is associated with higher risk of hip fracture in elderly human, and elderly women are more susceptible to hip fracture than aged men. Moreover, rats of the same gender will eliminate the effect posed by the gender difference and make the results more reliable. What is more, elder rats are sensitive to the deficiency of nutrients. Therefore, in the present study, we compared the metabolic properties of the liver in elderly female rats drinking purified water and tap water. These results will provide comprehensive information regarding the potential effects of low-mineral water on health.

## 2. Materials and Methods

### 2.1. Animal and Experimental Design

There were two types of water used in this experiment. The tap water was municipal drinking water in Chongqing, and it was treated with an activated carbon water purifier (100188CH purifier, AMWAY) before it was consumed. Bottled purified water was purchased from supermarket and was representative of the purified water available in the market because there are no differences in mineral composition. The water qualities of the two types of drinking water are shown in Table 1.

Twenty 10-month-old specific-pathogen-free (SPF) Sprague–Dawley female rats were obtained from the Animal Experimental Center of AMU (license number: SCXK-2017-0002). Rats were kept on a 12-h light/dark cycle in a temperature-controlled room maintained at 25 ± 1 °C with a relative humidity of 50 ± 5% and were habituated to the condition for 1 week prior to the treatment. All animal procedures were performed according to a protocol approved by the Institutional Animal Use and Care Committee of the Army Medical University (AMU) and carried out by individuals with appropriate licenses.

The rats were assigned to two groups randomly (*n* = 10 each group) and given tap water or purified water for 3 months. Group T stands for tap water group and group P for purified water group. The water was freshly prepared every day and was free from bacterial contamination. Rats had free access to food and water. The food was provided by the Experimental Animal Center of the Army Medical University (license number SCX-2007-018) and strictly followed the standard of GB14924-2010 in China for experimental animal feed nutrition [23]. The composition of the feed is shown in Supplementary Table S1. The food and water consumption was measured every day and the weight of rats every 7 days. After 3 months, 24-h urine samples of rats were collected using metabolism cages (one rat per cage). These rats were then anesthetized with sodium pentobarbital via i.p. injection. Blood was collected via heart puncture; livers were removed and immediately transferred into liquid nitrogen and then stored at −80 °C.

### 2.2. Measurement of Blood and Urine Samples

Blood and urine samples were analyzed with an automatic biochemical analyzer (Beckman-Coulter, Fullerton, CA, USA). Blood samples were analyzed to determine the lipid profile, liver function, kidney function, and electrolyte. Urine samples were analyzed to determine the electrolyte.

### 2.3. Metabolomics Analysis

We performed a metabolomics analysis with a Q300 Kit (Metabo-Profile, Shanghai, China). Liver samples were used to assess individual metabolites with ultra-performance liquid chromatography–tandem mass spectrometry (UPLC-MS/MS). The sample preparation and derivatization protocols were based on the method previously published, with minor modifications [24]. The details of sample preparation and derivatization protocols, as well as the UPLC-MS/MS instrument settings of the analysis, are shown in the Supplementary Materials. Internal standards were added to the test samples in order to monitor analytical variations during the entire sample preparation and analysis processes. The calibrators consisted of a blank sample (matrix sample processed without internal standard), a zero sample (matrix sample processed with internal standard), and a series of seven concentrations covering the expected range for the metabolites present in the specific biological samples.

### 2.4. Statistics

The raw data generated via UPLC-MS/MS were processed using the TMBQ software (v1.0, HMI, Shenzhen, China) to perform peak integration, calibration, and quantitation for each metabolite. The self-developed platform iMAP (v1.0, Metabo-Profile, Shanghai, China) was used for statistical analyses, including multi-dimensional statistics [principal component analysis (PCA), orthogonal partial least squares discriminant analysis (OPLS-DA)], univariate analysis (Student’s *t*-test or the Mann–Whitney U-test), pathway analysis, etc. PCA is an unsupervised modeling method commonly used to detect data outliers, clustering, and classification trends without a priori knowledge of the sample set. The whole process of PCA includes data normalization, calculating the covariance matrix, calculating eigenvectors and eigenvalues, selecting the principal component, and constructing the projection matrix. A variable of importance in the project (VIP) was obtained based on the OPLS-DA model. Metabolites with VIPs > 1 and *p*-values < 0.05 (univariate analyses) were considered significantly differentially expressed metabolites. The Z-score indicates the number of standard deviations by which an observation is above or below the mean of control group, the V-plot integrates the fold change, and the *p*-value indicates significantly different metabolites.

One-way ANOVA, followed by a least significant difference (LSD) test, was performed to compare the differences in routine blood and urine parameters. Data were presented as the mean ± SD. A *p*-value < 0.05 was considered statistically significant. Statistical analyses were conducted using SPSS 20.0.

## 3. Results

### 3.1. Data of Water Quality

The water qualities of the two types of drinking water are shown in Table 1. Purified water has much lower concentrations of minerals than tap water.

### 3.2. General Observations

**Figure 1:**
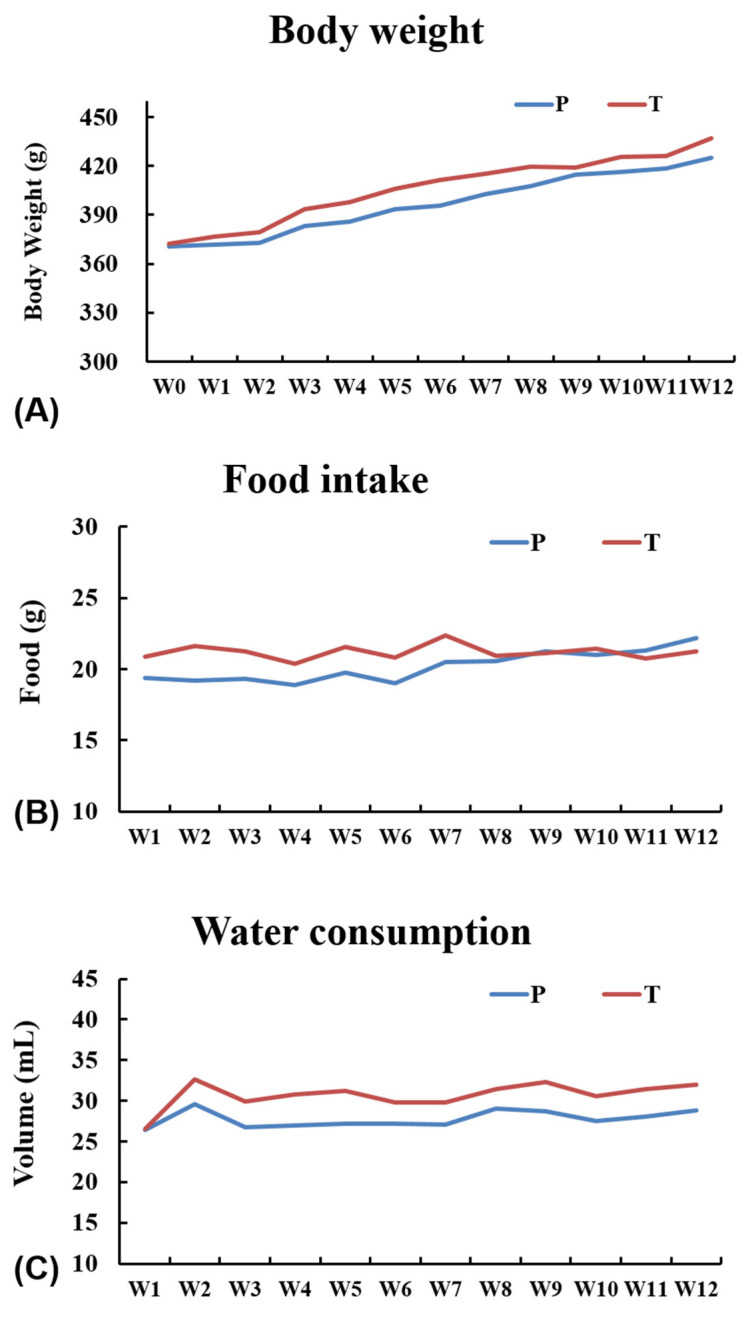
(**A**) Body weights of rats from week 0 to week 12. (**B**) Food intake of rats from week 1 to week 12. (**C**) Water consumption of rats from week 1 to week 12. P stands for the purified water group, and T stands for the tap water group.

The general appearances and physical conditions of rats were closely observed, and no obvious differences were noticed. There were no differences in body weight or water and food intake between groups throughout the experiment (Figure 1).

### 3.3. Serum and Urine Biochemistry

Blood samples were used to analyze lipids (triglyceride, total cholesterol, high-density lipoprotein, low-density lipoprotein, and the atherosclerotic index), electrolytes (K^+^, Na^+^, Ca^2+^, and Mg^2+^), liver function (alanine aminotransferase, aspartate transaminase, alkaline phosphatase, total protein, albumin, globulin, albumin/globulin, and prealbumin), and renal function (urea, creatinine, uric acid, retinol-binding protein). Urine samples were used to analyze electrolytes (K^+^, Na^+^, Ca^2+^, and Mg^2+^), urea, creatinine, and uric acid. There were no significant differences in all the aforementioned parameters between the two groups (Supplementary Tables S2 and S3).

### 3.4. Metabolic Profiling

**Figure 2:**
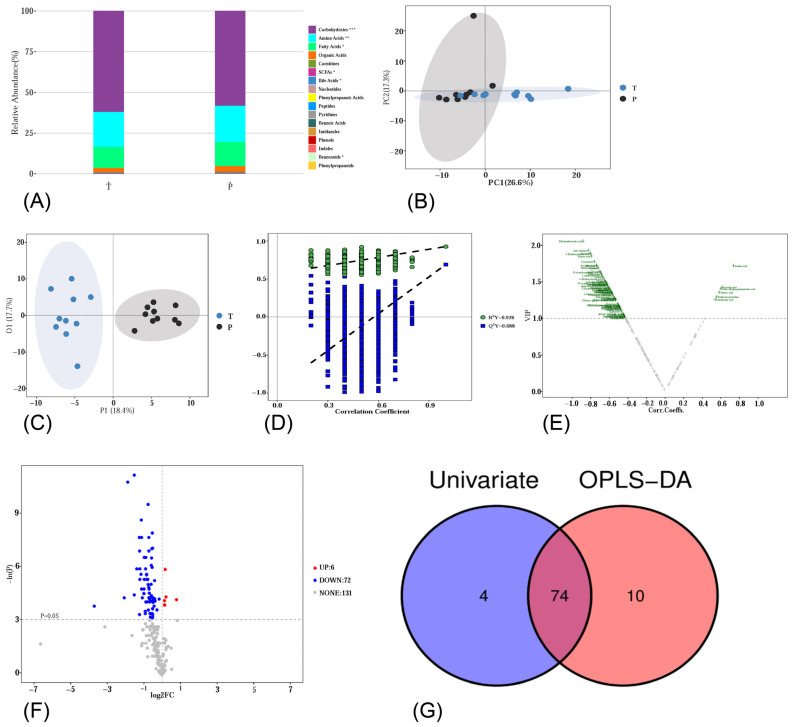
(**A**) Relative abundance of each metabolite class. (**B**) PCA score plot. (**C**) OPLS-DA 2D score plot. (**D**) Results of the permutation test. (**E**) Volcano plot of OPLS-DA model. The threshold value for differential metabolite selection is VIP > 1. (**F**) Volcano plot of univariate statistics. The threshold value for differential metabolite selection is *p* < 0.05 and |log_2_FC| > 0. (**G**) A Venn plot of differential metabolites from multi-dimensional statistics and univariate statistics. *, *p* < 0.05; **, *p* < 0.01; ***, *p* < 0.001.

Metabolomics analysis was performed using an UPLC-MS/MS system to determine the metabolite profile in rat liver samples. In total, 209 metabolites were detected. These metabolites were classified into different categories, including carbohydrates, amino acids, fatty acids, organic acids, carnitines, SCFAs, bile acids, nucleotides, phenylpropanoic acids, peptides, pyridines, benzoic acids, imidazoles, phenols, indoles, benzenoids, and phenylpropanoids. Figure 2A shows the relative abundance of each metabolite class. There are significant differences in carbohydrates, amino acids, fatty acids, SCFAs, bile acids, and benzenoids between group T and group P.

To further investigate the changes in liver metabolites, multivariate statistical analyses (unsupervised PCA and supervised OPLS-DA) were employed for metabolic data analysis. In the PCA score plot (Figure 2B), each point represents one individual liver sample of rats. A partial separation between group T and group P was observed along PC1, and this model explained 43.9% of the variability in the samples. OPLS-DA was further performed to show the differences between the two groups. As shown in Figure 2C, a distinct separation was observed. To validate the model against overfitting, a 1000-times permutation test was performed. As shown in Figure 2D, R^2^Y = 0.926 and Q^2^Y = 0.688, and the intercepts of Q^2^ regression lines were negative. These results suggested a low risk of overfitting and good predictive ability. A volcano plot of the OPLS-DA model is shown in Figure 2E. In group P, a total of 84 metabolites were significantly changed (Metabolites with VIP > 1), among which 6 metabolites significantly increased and 78 metabolites significantly decreased.

We also obtained differential metabolites using univariate statistical analysis (student’s *t*-test or the Mann–Whitney U-test, depending on the normality of data and homogeneity of variance). The volcano plot of univariate statistics is shown in Figure 2F, displaying the fold change (FC) and *p*-value of each metabolite. The threshold values for differential metabolite selection are *p* < 0.05 and |log_2_FC| > 0. In group P, 78 metabolites were significantly changed, with 6 metabolites increasing and 72 metabolites decreasing.

**Figure 3:**
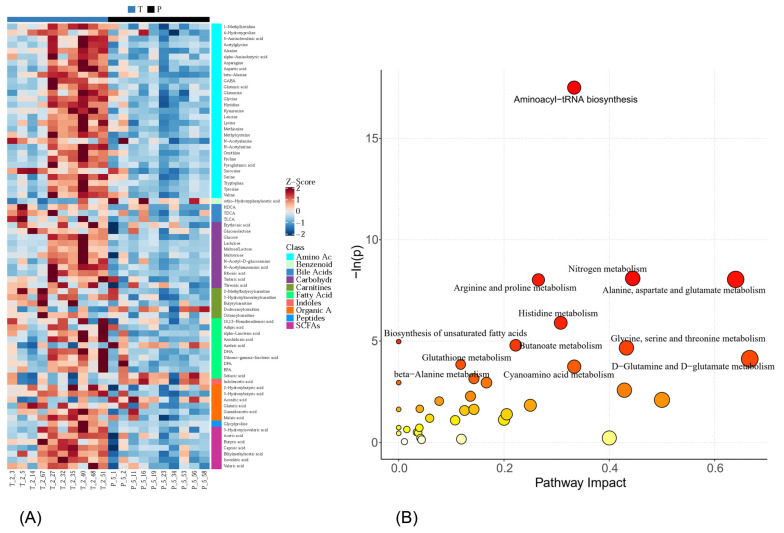
(**A**) Z Score plot of potential biomarkers; (**B**) metabolic pathway impact analysis via the RNO set. T stands for the tap water group, and P stands for the purified water group.

By identifying the intersection of the differential metabolites from univariate statistics and multivariate statistics, we found 74 significantly differentially expressed metabolites, including 29 amino acids, 11 carbohydrates, 10 fatty acids, 7 SCFAs, and 17 other biomolecules (Figure 2G). In group P, the levels of aconitic acid, azelaic acid, dodecanoylcarnitine, indoleacetic acid, sebacic acid, and ortho-hydroxyphenylacetic acid significantly increased in the liver, while the other 68 metabolites significantly decreased. The details of these 74 metabolites are summarized in Figure 3A and Table 2.

The significantly differentially expressed metabolites mentioned above were considered potential biomarkers. We further investigated the metabolic pathways of these 74 potential biomarkers using RNO sets. According to the metabolic pathway impact analysis, 8 pathways were significantly changed (*p* < 0.05, FDR < 0.1), namely aminoacyl–tRNA biosynthesis; nitrogen metabolism; alanine, aspartate, and glutamate metabolism; arginine and proline metabolism; histidine metabolism; the biosynthesis of unsaturated fatty acids; butanoate metabolism; glycine, serine, and threonine metabolism (Figure 3B and Table 3).

## 4. Discussion

There is an increasing trend around the world to drink low-mineral water. Long-term consumption of low-mineral water can lead to nutritional deficiencies, higher risk of cardiovascular disease, osteoporosis, certain neurodegenerative diseases, etc. However, the specific mechanism remains to be clarified. Metabolites are the final downstream products of protein translation and gene transcription. Metabolic abnormalities lead to the dysfunction of metabolic pathways and metabolite accumulation or deficiency, which are well-recognized hallmarks of diseases [25]. Metabolomics methods are useful to extract latent biochemical information about the characteristics of metabolites in complex organisms affected by environmental stresses and provide real biological endpoints [26]. This study was conducted to analyze the metabolic properties of the liver in rats drinking purified water using absolute quantitative metabolomics. A total of 74 significantly differentially expressed metabolites were identified: 6 metabolites significantly increased and 68 metabolites significantly decreased in rats drinking purified water. The 74 significantly differentially expressed metabolites were composed of 29 amino acids, 11 carbohydrates, 10 fatty acids, 7 SCFAs, and 17 other biomolecules.

Amino acids, the physical basis providing nitrogen, hydrocarbon skeletons, and sulfur to support life, are essential precursors for the synthesis of proteins, peptides, and low-molecular-weight substances with enormous physiological importance [27]. A sufficient supply of amino acids is a prerequisite for maintaining the optimal rate of protein synthesis [28]. In this study, all the 29 amino acids significantly decreased in rats drinking purified water, including 6 essential amino acids (histidine, leucine, lysine, methionine, tryptophan, and valine). As the adaptor decodes mRNA sequence into protein, the transfer RNAs (tRNAs) bring amino acids to the growing polypeptide chain at the ribosome and read the three base codons that define protein sequences [29]. Metabolic pathway impact analysis revealed that aminoacyl–tRNA biosynthesis was the top dysregulated metabolic pathway in the liver of rats drinking purified water. Among the 67 compounds in the aminoacyl–tRNA biosynthesis pathway observed in this study, 15 significantly decreased (Table 3). These results suggested that the aminoacyl–tRNA biosynthesis pathway was significantly downregulated in the liver of rats drinking purified water, which would limit protein synthesis and cell growth.

Nitrogen metabolism is the most basic process of material and energy metabolism in living organisms. Nitrogen is essential for the de novo synthesis of a variety of biomolecules including nucleotides, amino acids, polyamines, hexosamines, etc. [30]. Amino acids account for most of the biomass of proliferating cells and are a major reservoir for cellular nitrogen [30]. Glutamine and glutamate (glutamic acid), which are the major reservoirs of nitrogen in cells, are nitrogen donors for synthesizing many nitrogenous compounds. There were nine compounds in the nitrogen metabolism pathway, among which four (glutamic acid, glutamine, histidine, and glycine) significantly decreased in this study (Table 3). The results indicated a negative nitrogen balance in mice drinking purified water. Essential amino acids cannot be synthesized by the body and must be obtained from diet. In this study, all the rats had free access to the same food. There were no differences in food intake between the two groups throughout the experiment. What is more, no differences in the CREA, UA, and UREA of serum and urine were observed between the two groups. Thus, the absorption of amino acids may have reduced in rats drinking purified water. Taken together, drinking purified water may downregulate the absorption of amino acids and biosynthesis of aminoacyl–tRNA, leading to a negative nitrogen balance. Limitations of nitrogen availability in cells can disrupt the synthesis of proteins, nucleic acids, and other important nitrogen-containing compounds [30].

Amino acids also function as signal molecules and participate in the regulation of the metabolism of the body, such as protein synthesis [28]. In total, contents of 6 (aspartic acid, alanine, glutamic acid, GABA, glutamine, and asparagine) of the 24 metabolites in alanine, aspartate, and glutamate metabolism significantly decreased in this study (Table 3). Aspartic acid and glutamic acid are known as major excitatory neurotransmitters [31], while GABA is an inhibitory neurotransmitter [32]. The ratio of glutamate to GABA in the central nervous system, known as the excitatory–inhibitory (E/I) balance, modulates a wide range of cognitive and behavioral processes [33]. Perturbations to the E/I balance have been linked to the disruption of these processes in several psychiatric disorders [34], ranging from autism to schizophrenia [33]. Minerals, such as aluminum, silica, calcium, cadmium, and zinc, in drinking water could have an effect on cognitive aging [35]. The EPIDOS study showed that silica in drinking water may reduce the risk of developing Alzheimer’s disease (AD) in elderly women [35]. The PAQUID Study revealed a significant protective effect of high calcium and silica concentrations in drinking water on cognitive impairment [11,36]. Rats drinking purified water with a magnesium-deficient diet induced growth delay and reflex development stuntedness in F1-offspring [37]. Rats drinking filtered tap water with toxic contaminants removed and beneficial minerals retained bore offspring with better learning and memory abilities [38].

In total, 8 (glutamine, ornithine, aspartic acid, glutamic acid, proline, 4-hydroxyproline, guanidoacetic acid, and GABA) of the 44 metabolites were significantly decreased in arginine and proline metabolism in this study (Table 3). Proline, a non-essential proteinogenic amino acid, plays a multifaceted role in protein synthesis, redox balance, cell fate regulation, brain development, and other cellular and physiological processes [39]. Numerous studies have linked proline metabolism with ROS [40]. Proline synthesis and degradation are both highly redox-active processes [41]. Proline and proline metabolism can act as both ROS scavengers and producers [39]. Consequently, it is critical to balance proline levels and proline metabolic enzyme activities to achieve proline homeostasis for proper cellular functions [39].

For histidine metabolism, 4 (glutamic acid, histidine, aspartic acid, and 1-methylhistidine) out of 15 metabolites were significantly decreased in rats drinking purified water in this study (Table 3). Histidine is a dietary essential amino acid with unique roles in proton buffering, metal ion chelation, the scavenging of reactive oxygen and nitrogen species, erythropoiesis, and the histaminergic system [42]. Histidine can be used for protein synthesis, carnosine and anserine synthesis, and histamine synthesis [43]. Nowadays, histidine are investigated to prevent fatigue during strenuous exercise and for therapy in ageing-related disorders, metabolic syndrome, atopic dermatitis, ulcers, inflammatory bowel diseases, ocular diseases, and neurological disorders [42].

Fatty acids (FAs) are organic acids that are defined largely by the length and saturation of their aliphatic side chain. The side chains of animal FAs are classified into short chains, medium chains, long chains, and very long chains [44]. FAs also can be categorized as saturated, unsaturated, or polyunsaturated based on the number and presence of double bonds. FAs have diverse functions that range from structural “building blocks” of cell membranes to suppliers of energy and signaling molecules in cells [45]. Impaired uptake and metabolism of FAs have been implicated in several conditions, such as obesity-related insulin resistance and cardiovascular disease [44].

In this study, omega-3 polyunsaturated fatty acids (n-3 PUFAs), namely alpha-linolenic acid (ALA, 18:3n-3), eicosapentaenoic acid (EPA, 20:5n-3), docosahexaenoic acid, (DHA, 22:6n-3) and docosapentaenoic acid (DPA, 22:5n-3), were significantly reduced in the livers of rats drinking purified water. Meanwhile, arachidonic acid (AA, 20:4n-6) and dihomo-gamma-linolenic acid (DGLA, 20:3n-6) were reduced in rats drinking purified water. ALA, an essential fatty acid, can be converted to EPA, DPA, and DHA. The health benefits of n-3 PUFAs are numerous. n-3 PUFAs have been associated with improved development of the brain and vision in developing fetuses and reduced risk of CVD, obesity, metabolic syndrome, diabetes, arthritis, cognitive decline, and GI cancers [46].

Dietary n-6 PUFAs primarily include linoleic acid (LA, 18:2n-6) and AA. LA can be converted to DGLA, from which AA may be synthesized upon further desaturation [47]. Abundant evidence from prospective cohort studies and RCTs has shown that high n-6 PUFA intake plays an important role in the dietary prevention of CVD [48]. AA is a major component of mammalian cells and the direct precursor of bioactive lipid metabolites of eicosanoids. The abnormal metabolism of AA is closely related to the occurrence and development of many diseases, such as cardiovascular disease, inflammatory bowel disease, and asthma. Low AA levels affect sleep, elevate blood lipids, and cause fetal brain dysplasia [49]. DGLA has anti-inflammatory and antiproliferative properties [47]. Low levels of DGLA in serum have been related to poor outcomes in myocardial infarction (MI) patients [50].

SCFAs are saturated fatty acids with a chain length ranging from one to six carbon atoms, and they are the main metabolites produced via bacterial fermentation of dietary fiber in the gastrointestinal tract [51]. Acetate, propionate, and butyrate are the most abundant SCFAs [52]. SCFAs affect the occurrence and development of various diseases, such as type 2 diabetes, non-alcoholic fatty liver disease, inflammatory bowel disease, and colorectal cancer [53]. Furthermore, increasing evidence indicates the importance of SCFAs in regulating cardiovascular function. Butyrate and propionate can reduce blood pressure, improve ischemia/reperfusion injury, and decrease the risk of coronary artery disease and atherosclerosis. Acetate can also play a positive role in regulating hypertension and preventing atherosclerosis [54]. SCFAs exert their effects mainly through enhancing the intestinal barrier function, inhibiting the inflammatory response, promoting apoptosis, increasing the expression of G-protein-coupled receptors, affecting histone acetylation, and regulating immunity [53]. In this study, all of the detected SCFAs significantly decreased in rats drinking purified water (Table 2). Hence, long-term drinking of purified water may result in a reduction in SCFA-producing bacteria in the intestine, which will lead to lower concentrations of SCFAs. Lower expression of SCFAs would have a negative impact on health.

Carbohydrates are important macronutrients that provide energy and nutrients to the body, and they are categorized into simple sugars, complex carbohydrates, and glycoconjugates based on their structures [55]. Carbohydrates, predominantly glucose, are initially and most rapidly utilized during periods of energy shortage, which makes them the primary source of biological energy in living organisms [56]. It is critical to maintain glucose levels around 5.5 mM in the blood. There were 11 carbohydrates, including glucose, lactose, maltose, and maltotriose, that were significantly decreased in livers of rats drinking purified water in this study. Maltotriose, the second most abundant sugar in wort, is formed from the breakdown of complex sugars during mashing [57]. Maltose, a disaccharide produced from starch, has the same empirical formula as sucrose and lactose but differs from both in structure [58]. Lactose, maltose, and maltotriose can be easily utilized for energy. Glucose metabolism is tightly regulated to maintain human health and the homeostasis of glucose and energy production via gluconeogenesis and glycogenolysis [55]. In mammals, important sources that provide the carbons for gluconeogenesis are lactate, glycerol, and the amino acids alanine and glutamine [55]. The levels of alanine and glutamine significantly declined in liver of rats drinking purified water in this study. The declined alanine and glutamine may partially account for declines in gluconeogenesis, eventually resulting in lower levels of glucose. These results showed that long-term consumption of purified water disturbed energy metabolism in rats.

Mineral-rich drinking water is an important source of nutrients, such as calcium and magnesium. Compared with tap water, purified water has extremely low concentrations of minerals (Table 1). Long-term consumption of low-mineral water can lead to nutritional deficiencies, such as deficiencies of calcium, magnesium, and other essential elements [6]. Calcium is the main mineral in the body. Over 99% of total body calcium is found in bones and teeth, functioning as a key structural element. The remaining body calcium takes part in metabolism, serving as a signal for vital physiological processes, including vascular contraction, blood clotting, muscle contraction, and nerve transmission [4]. Magnesium is a cofactor in more than 300 enzyme systems that regulate diverse biochemical reactions in the body, including protein synthesis, muscle and nerve transmission, neuromuscular conduction, signal transduction, blood glucose control, and blood pressure regulation [59]. Some enzymes including Na^+^/K^+^-ATPase, hexokinase, creatine kinase, protein kinase, and cyclases are magnesium-dependent enzymes [59]. Aminoacyl–tRNA synthetases (AaRSs) aminoacylate tRNA molecules with their cognate amino acid and provide substrates for protein biosynthesis. Magnesium is essential for the AaRSs’ activity [60] and is an important cofactor for the normal activities of desaturases and elongases in endogenous FAs synthesis [61]. Therefore, inadequate intake of calcium, magnesium, and other nutrients from purified water may account for the metabolic disturbance of liver in rats.

There are some limitations to this study. First, we only explored the liver metabolic alterations in aged female rats after drinking purified water over a period of three months. It is necessary to explore the metabolic alterations in both male and female rats over a longer period. Second, we did not explore the underlying molecular mechanism, which will be focus of our future research. Finally, empirical data from human subjects are also needed to further verify the findings.

## 5. Conclusions

In summary, this study was the first experiment that utilized a metabolomics-based strategy to explore the health effects of purified water. After the rats were exposed to purified water for 3 months, 74 metabolites (6 increased and 68 decreased) and 8 metabolic pathways were significantly changed in purified water group. These changes suggested that the consumption of purified water induced negative nitrogen balance, reduced the expression of some polyunsaturated fatty acids and short-chain fatty acids, and disturbed energy metabolism in rats. These metabolic disturbances may underlie low-mineral-water-associated health risks. It is urgent to pay more attention to the health risks of low-mineral water.

## Figures and Tables

**Table 1:** Water qualities of the two types of drinking water.

Index	pH	TDS g/m^3^	TH g/m^3^	HCO_3_^−^ g/m^3^	Ca^2+^ g/m^3^	Mg^2+^ g/m^3^	K^+^ g/m^3^	Na^+^ g/m^3^	H_2_SiO_3_ g/m^3^
Tap water	8.32	333.87	184.64	166.99	50.4	14.9	5.12	14.16	5.44
Purified water	6.33	3.63	0.43	0	0.17	0.08	0.04	0.33	0

TDS, total dissolved solid; TH, total hardness.

**Table 2:** Differentially expressed metabolites in liver of rats drinking purified water.

Class	HMDB	KEGG	Metabolite	*p*-Value	FDR	Fold Change	VIP	Trend
Amino Acids	HMDB0000182	C00047	Lysine	2.652 × 10^−3^	0.029	0.687	1.56	↓
Amino Acids	HMDB0000177	C00135	Histidine	9.229 × 10^−4^	0.018	0.679	1.72	↓
Amino Acids	HMDB0000214	C00077	Ornithine	3.546 × 10^−2^	0.102	0.636	1.14	↓
Amino Acids	HMDB0000641	C00064	Glutamine	1.768 × 10^−2^	0.065	0.768	1.20	↓
Amino Acids	HMDB0000148	C00025	Glutamic acid	1.469 × 10^−2^	0.064	0.616	1.46	↓
Amino Acids	HMDB0000271	C00213	Sarcosine	5.196 × 10^−3^	0.039	0.563	1.39	↓
Amino Acids	HMDB0000056	C00099	beta-Alanine	1.050 × 10^−3^	0.018	0.428	1.93	↓
Amino Acids	HMDB0000161	C00041	Alanine	7.578 × 10^−5^	0.005	0.591	1.89	↓
Amino Acids	HMDB0000112	C00334	GABA	8.931 × 10^−3^	0.053	0.461	1.45	↓
Amino Acids	HMDB0000187	C00065	Serine	1.295 × 10^−2^	0.064	0.670	1.35	↓
Amino Acids	HMDB0002108	NA	Methylcysteine	4.871 × 10^−4^	0.013	0.461	1.69	↓
Amino Acids	HMDB0000158	C00082	Tyrosine	1.150 × 10^−2^	0.063	0.502	1.34	↓
Amino Acids	HMDB0000168	C00152	Asparagine	3.886 × 10^−3^	0.035	0.558	1.45	↓
Amino Acids	HMDB0000684	C00328	Kynurenine	2.165 × 10^−5^	0.002	0.273	1.77	↓
Amino Acids	HMDB0000191	C00049	Aspartic acid	4.326 × 10^−2^	0.117	0.697	1.36	↓
Amino Acids	HMDB0000766	NA	N-Acetyalanine	5.196 × 10^−3^	0.039	0.581	1.39	↓
Amino Acids	HMDB0000123	C00037	Glycine	3.805 × 10^−4^	0.013	0.691	1.68	↓
Amino Acids	HMDB0000162	C00148	Proline	2.879 × 10^−3^	0.029	0.432	1.51	↓
Amino Acids	HMDB0000532	NA	Acetylglycine	1.553 × 10^−2^	0.064	0.753	1.36	↓
Amino Acids	HMDB0002931	NA	N-Acetylserine	3.546 × 10^−2^	0.102	0.529	1.36	↓
Amino Acids	HMDB0000883	C00183	Valine	1.615 × 10^−2^	0.064	0.719	1.27	↓
Amino Acids	HMDB0000267	C01879	Pyroglutamic acid	6.841 × 10^−3^	0.047	0.662	1.46	↓
Amino Acids	HMDB0000696	C00073	Methionine	1.854 × 10^−2^	0.065	0.568	1.27	↓
Amino Acids	HMDB0000687	C00123	Leucine	8.931 × 10^−3^	0.053	0.610	1.32	↓
Amino Acids	HMDB0000929	C00078	Tryptophan	1.469 × 10^−2^	0.064	0.633	1.09	↓
Amino Acids	HMDB0000001	C01152	1-Methylhistidine	8.977 × 10^−4^	0.018	0.701	1.59	↓
Amino Acids	HMDB0000452	C02356	alpha-Aminobutyric acid	3.886 × 10^−3^	0.035	0.450	1.44	↓
Amino Acids	HMDB0001149	C00430	5-Aminolevulinic acid	6.915 × 10^−3^	0.047	0.590	1.32	↓
Amino Acids	HMDB0000725	C01157	4-Hydroxyproline	5.550 × 10^−3^	0.040	0.752	1.49	↓
Benzenoids	HMDB0000669	C05852	ortho-Hydroxyphenylacetic acid	1.628 × 10^−2^	0.064	1.722	1.40	↑
Bile Acids	HMDB0000896	C05463	TDCA	4.498 × 10^−2^	0.121	0.696	1.27	↓
Bile Acids	HMDB0000722	C02592	TLCA	1.130 × 10^−2^	0.063	0.616	1.26	↓
Bile Acids	HMDB0000733	NA	HDCA	2.352 × 10^−2^	0.076	0.077	1.46	↓
Carbohydrates	HMDB0000943	C01620	Threonic acid	3.723 × 10^−2^	0.105	0.702	1.15	↓
Carbohydrates	HMDB0000150	C00198	Gluconolactone	1.902 × 10^−2^	0.065	0.712	1.42	↓
Carbohydrates	HMDB0000613	NA	Erythronic acid	1.774 × 10^−2^	0.064	0.661	1.37	↓
Carbohydrates	HMDB0000230	C00270	N-Acetylneuraminic acid	2.879 × 10^−3^	0.029	0.534	1.63	↓
Carbohydrates	HMDB0000122	C00221	Glucose	4.871 × 10^−4^	0.013	0.606	1.64	↓
Carbohydrates	HMDB0000740	C07064	Lactulose	2.879 × 10^−3^	0.029	0.383	1.25	↓
Carbohydrates	NA	NA	Maltose/Lactose	4.871 × 10^−4^	0.013	0.424	1.15	↓
Carbohydrates	HMDB0001262	C01835	Maltotriose	2.364 × 10^−3^	0.029	0.664	1.61	↓
Carbohydrates	HMDB0000215	C00140	N-Acetyl-D-glucosamine	8.931 × 10^−3^	0.053	0.504	1.48	↓
Carbohydrates	HMDB0000956	C00898	Tartaric acid	8.127 × 10^−3^	0.053	0.604	1.16	↓
Carbohydrates	HMDB0000867	C01685	Ribonic acid	1.469 × 10^−2^	0.064	0.239	1.50	↓
Carnitines	HMDB0002013	C02862	Butyrylcarnitine	1.245 × 10^−2^	0.064	0.349	1.29	↓
Carnitines	HMDB0000378	NA	2-Methylbutyroylcarnitine	1.505 × 10^−3^	0.022	0.509	1.33	↓
Carnitines	NA	NA	3-Hydroxylisovalerylcarnitine	1.616 × 10^−2^	0.064	0.739	1.17	↓
Carnitines	HMDB0000791	C02838	Octanoylcarnitine	1.578 × 10^−2^	0.064	0.897	1.13	↓
Carnitines	HMDB0002250	NA	Dodecanoylcarnitine	2.223 × 10^−2^	0.074	1.107	1.29	↑
Fatty Acids	HMDB0000784	C08261	Azelaic acid	2.959 × 10^−3^	0.029	1.130	1.72	↑
Fatty Acids	HMDB0000792	C08277	Sebacic acid	1.727 × 10^−2^	0.065	1.099	1.35	↑
Fatty Acids	HMDB0000448	C06104	Adipic acid	1.469 × 10^−2^	0.064	0.708	1.12	↓
Fatty Acids	HMDB0001388	C06427	alpha-Linolenic acid	1.469 × 10^−2^	0.064	0.609	1.25	↓
Fatty Acids	NA	NA	10,13-Nonadecadienoic acid	2.920 × 10^−2^	0.090	0.597	1.18	↓
Fatty Acids	HMDB0001999	C06428	EPA	5.196 × 10^−3^	0.039	0.433	1.40	↓
Fatty Acids	HMDB0001043	C00219	Arachidonic acid	1.150 × 10^−2^	0.063	0.504	1.42	↓
Fatty Acids	HMDB0002925	C03242	Dihomo-gamma-linolenic acid	3.546 × 10^−2^	0.102	0.635	1.25	↓
Fatty Acids	HMDB0002183	C06429	DHA	1.469 × 10^−2^	0.064	0.475	1.50	↓
Fatty Acids	HMDB0006528	C16513	DPA	1.854 × 10^−2^	0.065	0.626	1.02	↓
Indoles	HMDB0000197	C00954	Indoleacetic acid	2.191 × 10^−2^	0.074	1.097	1.25	↑
Organic Acids	HMDB0000661	C00489	Glutaric acid	2.890 × 10^−2^	0.090	0.824	1.26	↓
Organic Acids	HMDB0000072	C02341	Aconitic acid	1.398 × 10^−2^	0.064	1.164	1.42	↑
Organic Acids	HMDB0000128	C00581	Guanidoacetic acid	5.196 × 10^−3^	0.039	0.510	1.47	↓
Organic Acids	HMDB0000357	C01089	3-Hydroxybutyric acid	1.050 × 10^−3^	0.018	0.609	1.88	↓
Organic Acids	HMDB0000008	C05984	2-Hydroxybutyric acid	4.014 × 10^−3^	0.035	0.554	1.54	↓
Organic Acids	HMDB0000176	C01384	Maleic acid	2.349 × 10^−2^	0.076	0.740	1.17	↓
Peptides	HMDB0000721	NA	Glycylproline	1.854 × 10^−2^	0.065	0.542	1.00	↓
SCFAs	HMDB0000042	C00033	Acetic acid	2.711 × 10^−2^	0.086	0.699	1.39	↓
SCFAs	HMDB0000754	NA	3-Hydroxyisovaleric acid	3.115 × 10^−2^	0.094	0.528	1.41	↓
SCFAs	HMDB0000039	C00246	Butyric acid	1.835 × 10^−4^	0.010	0.456	1.85	↓
SCFAs	HMDB0000535	C01585	Caproic acid	1.609 × 10^−2^	0.064	0.625	1.39	↓
SCFAs	HMDB0002176	C18319	Ethylmethylacetic acid	1.462 × 10^−5^	0.002	0.350	2.06	↓
SCFAs	HMDB0000718	C08262	Isovaleric acid	1.508 × 10^−3^	0.022	0.542	1.72	↓
SCFAs	HMDB0000892	C00803	Valeric acid	1.556 × 10^−3^	0.022	0.674	1.63	↓

Variable of importance in the project (VIP) was obtained using OPLS-DA with a threshold of 1.0. *p*-value was calculated using Student’s *t*-test or Mann-Whitney U-test, depending on the normality of data and homogeneity of variance. ↓, decrease. ↑, increase. HMDB, Human Metabolome Database. KEGG, Kyoto Encyclopedia of Genes and Genomes. TDCA, taurodeoxycholic acid. TLCA, taurolithocholic acid. HDCA, hyodeoxycholic acid. GABA, γ-aminobutyric acid. EPA, eicosapentaenoic acid. DHA, docosahexaenoic acid. DPA, docosapentaenoic acid.

**Table 3:** Significantly changed pathways in livers of rats drinking purified water.

	Total in Pathway	Hits	Raw P	FDR	Impact	Enriched Compounds	KEGG Link
Aminoacyl–tRNA biosynthesis	67	15	2.49 × 10^−8^	2.02 × 10^−6^	0.333	Asparagine, Histidine, Glutamine, Glycine, Aspartic acid, Serine, Methionine, Valine, Alanine, Lysine, Leucine, Tryptophan, Tyrosine, Proline, Glutamic acid	http://www.genome.jp/kegg-bin/show_pathway?rno00970/C00152%09DeepSkyBlue/C00135%09DeepSkyBlue/C00064%09DeepSkyBlue/C00037%09DeepSkyBlue/C00049%09DeepSkyBlue/C00065%09DeepSkyBlue/C00073%09DeepSkyBlue/C00183%09DeepSkyBlue/C00041%09DeepSkyBlue/C00047%09DeepSkyBlue/C00123%09DeepSkyBlue/C00078%09DeepSkyBlue/C00082%09DeepSkyBlue/C00148%09DeepSkyBlue/C00025%09DeepSkyBlue (accessed on 18 May 2024)
Nitrogen metabolism	9	4	3.05 × 10^−4^	6.64 × 10^−3^	0.444	Glutamic acid, Glutamine, Histidine, Glycine	http://www.genome.jp/kegg-bin/show_pathway?rno00910/C00025%09DeepSkyBlue/C00064%09DeepSkyBlue/C00135%09DeepSkyBlue/C00037%09DeepSkyBlue (accessed on 18 May 2024)
Alanine, aspartate, and glutamate metabolism	24	6	3.21 × 10^−4^	6.64 × 10^−3^	0.640	Aspartic acid, Alanine, Glutamic acid, GABA, Glutamine, Asparagine	http://www.genome.jp/kegg-bin/show_pathway?rno00250/C00049%09DeepSkyBlue/C00041%09DeepSkyBlue/C00025%09DeepSkyBlue/C00334%09DeepSkyBlue/C00064%09DeepSkyBlue/C00152%09DeepSkyBlue (accessed on 18 May 2024)
Arginine and proline metabolism	44	8	3.28 × 10^−4^	6.64 × 10^−3^	0.265	Glutamine, Ornithine, Aspartic acid, Glutamic acid, Proline, 4-Hydroxyproline, Guanidoacetic acid, GABA	http://www.genome.jp/kegg-bin/show_pathway?rno00330/C00064%09DeepSkyBlue/C00077%09DeepSkyBlue/C00049%09DeepSkyBlue/C00025%09DeepSkyBlue/C00148%09DeepSkyBlue/C01157%09DeepSkyBlue/C00581%09DeepSkyBlue/C00334%09DeepSkyBlue (accessed on 18 May 2024)
Histidine metabolism	15	4	2.73 × 10^−3^	4.42 × 10^−2^	0.308	Glutamic acid, Histidine, Aspartic acid, 1-Methylhistidine	http://www.genome.jp/kegg-bin/show_pathway?rno00340/C00025%09DeepSkyBlue/C00135%09DeepSkyBlue/C00049%09DeepSkyBlue/C01152%09DeepSkyBlue (accessed on 18 May 2024)
Biosynthesis of unsaturated fatty acids	42	6	6.91 × 10^−3^	9.32 × 10^−2^	0	DPA, Arachidonic acid, Dihomo-gamma-linolenic acid, DHA, EPA, alpha-Linolenic acid	http://www.genome.jp/kegg-bin/show_pathway?rno01040/C16513%09DeepSkyBlue/C00002%09DeepSkyBlue/C03242%09DeepSkyBlue/C06429%09DeepSkyBlue/C06428%09DeepSkyBlue/C06427%09DeepSkyDeep (accessed on 18 May 2024)
Butanoate metabolism	20	4	8.27 × 10^−3^	9.45 × 10^−2^	0.222	3-Hydroxybutyric acid, GABA, Glutamic acid, Butyric acid	http://www.genome.jp/kegg-bin/show_pathway?rno00650/C01089%09DeepSkyBlue/C00003%09DeepSkyBlue/C00025%09DeepSkyBlue/C00246%09DeepSkyBlue (accessed on 18 May 2024)
Glycine, serine, and threonine metabolism	32	5	9.34 × 10^−3^	9.45 × 10^−2^	0.432	Serine, Guanidoacetic acid, Glycine, Sarcosine, 5-Aminolevulinic acid	http://www.genome.jp/kegg-bin/show_pathway?rno00260/C00065%09DeepSkyBlue/C00581%09DeepSkyBlue/C00037%09DeepSkyBlue/C00213%09DeepSkyBlue/C00430%09DeepSkyBlue (accessed on 18 May 2024)

## Data Availability

Data are contained within the article and Supplementary Materials.

## Supplementary Materials

The following supporting information can be downloaded via this link: https://www.mdpi.com/article/10.3390/metabo14050289/s1, Sample preparation and derivatization protocols; Instrument settings of the UPLC-MS/MS analysis; Supplementary Table S1. Composition of the feed; Supplementary Table S2. Biochemical parameters in serum; Supplementary Table S3. Biochemical parameters in urine.
