# Supplementary material for: Long-Term Consumption of Purified Water Altered Amino Acid, Fatty Acid and Energy Metabolism in Livers of Rats
Source: Metabolites. 2024 May 19;14(5):289. doi: 10.3390/metabo14050289 (PMC11122726; doi:10.3390/metabo14050289)
Supplement: Supplementary file 1 [file metabolites-14-00289-s001.zip › metabolites-3003647-supplementary.pdf]

Supplementary data

Long-term consumption of purified water altered amino acid, fatty acid and energy metabolism in liver of rats

WANG jia[1], QIU zhiqun[2], ZENG hui[2], TAN yao[2], HUANG yujing[2], LUO jiaohua[2*], SHU weiqun[2*]

[1]Department of Medical English, College of Basic Medicine, Army Medical University (Third Military Medical University), Chongqing, 400038, China

[2]Department of Environmental Hygiene, College of Preventive Medicine, Army Medical University (Third Military Medical University), Chongqing, 400038, China

***Correspondence: ljh978@tmmu.edu.cn; Telephone: +86-23-68771559. weiqunshu@tmmu.edu.cn; Telephone: +86-23-68771559.**

Table of contents

1. Supplementary materials and methods

1.1 Sample preparation and derivatization protocols

1.2 Instrument settings of the UPLC-MS/MS analysis

2. Supplementary Tables

2.1 Supplementary Table S1. Composition of the feed

2.2 Supplementary Table S2. Biochemical parameters in serum

2.3 Supplementary Table S3. Biochemical parameters in urine

1. Supplementary materials and methods

1.1 Sample preparation and derivatization protocols

Each tissue sample (~10mg) that was harvested and stored in an Eppendorf Safelock microcentrifuge tube was mixed with 10 pre-chilled zirconium oxide beads and 20μL of deionized water. The sample was homogenated for 3 minutes and 120μL of Methanol containing internal standard was added to extract the metabolites. The sample was homogenated for another 3 minutes and then centrifuged at 18000g for 20 minutes. Then the supernatant was transferred to a 96-well plate. The following procedures were performed on an Eppendorf epMotion Workstation (Eppendorf Inc., Humburg, Germany). 20μL of freshly prepared derivative reagents was added to each well. The plate was sealed and the derivatization was carried out at 30°C for 60 min. After derivatization, the sample was evaporated for 2h. 330μL of ice-cold 50% methanol solution was added to reconstitute the sample. Then the plate was stored at -20°C for 20 minutes and followed by 4000g centrifugation at 4°C for 30 minutes. 135μL of supernatant was transferred to a new 96-well plate with 10μL internal standards in each well. Serial dilutions of derivatized stock standards were added to the left wells. Finally the plate was sealed for LC-MS analysis.

1.2 **Instrument settings of the UPLC-MS/MS analysis**

An ultra-performance liquid chromatography coupled to tandem mass spectrometry (UPLC-MS/MS) system (ACQUITY UPLC-Xevo TQ-S, Waters Corp., Milford, MA, USA) was used to quantitate all targeted metabolites in this project. The optimized instrument settings are briefly described below. The instrument performance optimization and routine maintenance were performed every week.

UPLC-MS/MS instrument settings	
UPLC	
Column	ACQUITY UPLC BEH C18 1.7 μM VanGuard pre-column (2.1×5 mm) and ACQUITY UPLC BEH C18 1.7 μM analytical column (2.1 × 100 mm)
Column Temp. (°C)	40
Sample Manager Temp. (°C)	10
Mobile Phases	A=water with 0.1% formic acid; and B=acetonitrile/IPA (70:30)
Gradient Conditions	0-1 min (5% B), 1-11min (5%-78% B), 11-13.5 min (78%-95% B), 13.5-14 min (95%-100% B), 14-16 min (100% B), 16-16.1 min (100%-5% B), 16.1-18 min (5% B).

Flow Rate (mL/min)	0.40
Injection Vol. (μL)	5.0
MASS SPECTROMETER	
Capillary (Kv)	1.5 (ESI+), 2.0 (ESI-)
Source Temp (°C)	150
Desolvation Temp (°C)	550
Desolvation Gas Flow (L/hr)	1000

2. Supplementary tables

Supplementary Table S1. Composition of the feed (per Kg)

Gradient	Weight	Gradient	Weight	Gradient	Weight	Gradient	Weight
Crude protein	≥200g	Histidine	≥5.5g	Vitamink	≥5.0mg	Vitaminc	-
Crude fat	≥40g	Tryptophan	≥2.5g	Vitaminb1	≥13mg	Magnesium	≥2.0g
Crude fiber	≤50g	Phenylalanine + Tyrosine	≥13g	Vitaminb2	≥12mg	Potassium	5.0g
Crude ash	≤80g	Threonine	≥8.8g	Vitaminb6	≥12mg	Sodium	≥2.0g
Moisture content	≤100g	Leucine	≥17.6g	Niacin	≥60mg	Ferrum	≥120mg
Calcium	10-18g	Isoleucine	≥10.3g	Pantothenic acid	≥24mg	Manganese	≥75mg
Phosphorus	6-12g	Valine	≥11.7g	Folate	≥6.0mg	Copper	≥10mg
Lysine	≥13.2g	Vitamin A	≥14000IU	Biotin	≥0.2mg	Zinc	≥30mg
Methionine + Cystine	≥7.8g	Vitamin D	≥1500IU	Vitamin B12	≥0.022mg	Iodine	≥0.5mg
Arginine	≥11g	Vitamin E	≥120IU	Choline	≥1250mg	Selenium	0.1-0.2mg

The composition of the feed was strictly followed the standard of GB14924-2010 in China for experimental animal feed nutrition.

Supplementary Table S2. Biochemical parameters in serum (Mean ± SD)

	Group T	Group P	*P* value
Triglyceride (mmol/L)	2.97±1.27	3.37±2.42	0.63
Total cholesterol (mmol/L)	3.23±0.71	3.00±0.72	0.45
High density lipoprotein, HDL (mmol/L)	0.72±0.16	0.68±0.11	0.46
Low density lipoprotein, LDL (mmol/L)	0.38±0.10	0.37±0.12	0.76
AI	3.54±0.76	3.43±0.95	0.78
K^+ (mmol/L)	10.03±1.44	9.71±1.34	0.57
Na^+ (mmol/L)	135.49±1.55	135.84±1.36	0.64
Ca^{2+} (mmol/L)	2.74±0.15	2.69±0.14	0.33
Mg^{2+} (mmol/L)	1.24±0.14	1.21±0.09	0.57

CaAd (mmol/L)	2.84±0.13	2.78±0.12	0.27
Alkaline phosphatase (U/L)	67.31±31.04	72.01±30.45	0.77
Urea (mmol/L)	7.2±1.44	7.18±1.90	0.98
Creatinine (μmol/L)	42.93±6.16	42.22±6.72	0.8
Uric acid (μmol/L)	113.72±47.51	100.66±40.93	0.39
Retinol-binding protein (mg/L)	3.00±0.58	2.80±0.63	0.43
Alanine aminotransferase (IU/L)	94.91±43.52	81.69±26.67	0.54
Aspartate transaminase (IU/L)	177.7±113.78	185.41±99.85	0.89
Total protein (g/L)	79.18±5.86	77.36±4.81	0.41
Albumin (g/L)	35.47±2.51	35.22±2.84	0.81
Globulin (g/L)	43.72±4.62	42.14±3.62	0.35
Albumin/globulin	0.82±0.09	0.84±0.09	0.52
Prealbumin (mg/L)	3.08±0.86	2.80±0.92	0.48

AI, Atherosclerotic index = (TC-HDL)/HDL; CaAd, Albumin-adjusted Ca^{2+}. P stands for purified water group, and T for tap water group. Values are mean ± SD, n = 10.

Supplementary Table S3. Biochemical parameters in urine (Mean ± SD)

	Group T	Group P	*P* value
K^+ (mmol/L)	68.64±14.01	62.56±14.93	0.981
Na^+ (mmol/L)	49.70±19.3	42.94±17.2	0.992
Ca^{2+} (mmol/L)	3.14±2.04	3.71±1.83	0.448
Mg^{2+} (mmol/L)	2.21±2.12	1.63±1.88	0.413
UREA (mmol/L)	340.53±160.60	328.45±155.14	0.827
CREA (μmol/L)	3004.83±1437.10	3192.87±840.62	1.0
UA (μmol/L)	317.32±172.41	328.69±166.67	1.0
Oxalate (mmol/L)	0.18±0.09	0.13±0.08	0.184
Citrate (mmol/L)	1.09±0.22	1.08±0.19	0.923

P stands for purified water group, and T for tap water group.
Values are mean ± SD, n = 10.
